# Editorial: Seeking equal opportunities and safe environments: research from a gender perspective

**DOI:** 10.3389/fpsyg.2025.1530113

**Published:** 2025-01-27

**Authors:** Alba García-Cid, Clarissa Pepe-Ferreira, Patricia García-Leiva, Raquel Diniz, Lourdes Villardón-Gallego

**Affiliations:** ^1^Department of Psychology, Health Sciences Faculty, University of Deusto, Bilbao, Spain; ^2^Posgraduate Program in Social Memory, UNIRIO, Rio de Janeiro State Federal University, Rio de Janeiro, Brazil; ^3^Department of Social Psychology, Social Work and Social Services and Social Anthropology, Faculty of Psychology and Speech Therapy, University of Malaga, Málaga, Spain; ^4^Department of Psychology, PPgPsi - UFRN, Federal University of Rio Grande do Norte, Natal, Rio Grande do Norte, Brazil; ^5^Department of Education, Faculty of Education and Sports, University of Deusto, Bilbao, Spain

**Keywords:** gender based violence, educational preventive programmes, cultural differences, dehumanization, institutional actions, gender equity, socioeconomic disparities

The articles published in this Editorial collectively underscore key patterns in the study and prevention of gender-based violence and the pursuit of equal opportunities in society. Across various settings—academic institutions, workplaces, and family environments—these studies reveal that societal norms and institutional actions play crucial roles in either perpetuating or mitigating gender disparities and biases. There are some common and strong points:

1. **Dehumanization and gender norms**: research by Tanriverdi et al. highlights how nonconforming women experience mechanistic dehumanization, revealing subtle forms of marginalization in male-dominated environments. This underscores the importance of dismantling rigid gender expectations to foster inclusivity.
2. **Policy and institutional gaps**: studies by dos Santos Barbosa et al. and Hurtado-Reina et al. show how educational institutions and workplaces often lack policies addressing violence and representation, which can enable gender-based harassment or overlook female academic contributions. Both studies advocate for robust, zero-tolerance policies and greater visibility of women's work to achieve equity.
3. **Cultural and socioeconomic factors in violence**: Nwankpa et al. and Karamitanha et al. examine cultural beliefs and socioeconomic disparities, showing that gender biases within households and among partners contribute to violence and inequality. Their findings emphasize the need for targeted educational and economic interventions to transform attitudes about gender roles and empower women.
4. **Impact of gender-based violence**: the work of Athanasiades et al., Ganesan and Gopalakrishnan, and Chela-Alvarez et al. captures the psycho-emotional toll of harassment and abuse on women, emphasizing resilience and the normalization of harassment in precarious work environments. These studies highlight the need for comprehensive interventions that address workplace and academic settings to protect women's wellbeing.
5. **Promoting gender equity and inclusivity**: studies by Bae and Jeong, Wen et al., and Qin et al. highlight effective strategies to reduce gender bias and foster gender equity in STEM and other fields, such as brief video interventions and supportive university environments. These studies emphasize the importance of promoting educational opportunities and workplace equity to enhance women's self-perceived employability and reduce bias.

These findings collectively call for comprehensive policy reforms, educational programs, and cultural shifts to reduce gender-based violence and foster environments that value diversity and equality. They also highlight that the journey toward gender equity requires institutions to take proactive measures and implement targeted interventions to support marginalized and vulnerable groups in diverse social contexts.

The following is a brief summary of each of the articles published in this editorial, highlighting their key findings and proposals.

Tanriverdi et al.'s study examines how women who do not conform to gender expectations in tone of voice, occupation, and appearance may be subject to dehumanization. A between-groups factorial experiment was conducted in which participants evaluated a target woman with congruent or incongruent combinations of tone of voice, occupation, and appearance. Results showed that women with an incongruent tone of voice and occupation relative to their gender were more dehumanized, both mechanistically and animalistically. However, the interaction between tone of voice, type of occupation, and appearance was only significant for mechanistic dehumanization. This study reveals that women who deviate from gender norms in tone of voice and occupation face higher levels of mechanistic dehumanization, perceived as lacking warmth and traditional human qualities. These biases suggest that gender nonconformity leads to subtle marginalization, particularly in male-dominated settings, potentially impacting women's social and professional experiences.

The article from Royo et al. explores the limitations of traditional surveys in capturing the complexities of sex, gender, and sexual orientation. The authors argue that conventional binary categories inadequately reflect the diversity of identities, leading to the underrepresentation of marginalized groups. They propose an inclusive framework for survey design that incorporates a broader spectrum of gender identities and sexual orientations, aiming to improve data accuracy and inform policies that better address the needs of diverse populations.

dos Santos Barbosa et al., adopt a multi-level perspective to analyze factors contributing to violence against female students in university settings. Through in-depth interviews with 20 university students, both female victims and male aggressors, 41 analytical codes were identified, revealing three main categories: (1) Institutional Actions and Omissions: the lack of clear policies and institutional inaction in response to violence reports were found to foster an environment where such behaviors can proliferate. (2) Characteristics of Aggressors: some aggressors exhibited a predisposition toward violence, a sense of efficacy in their actions, and were influenced by their peer groups, which reinforced aggressive behaviors. (3) Dichotomy in Perception of Victims: women were viewed along a spectrum from vulnerable to strong, affecting their willingness to report incidents and seek support. The authors conclude that it is essential for universities to implement effective policies, promote a zero-tolerance culture toward gender-based violence, provide adequate support for victims, and establish reeducation programs for aggressors.

Nwankpa et al., investigates the attitudes of male and female household heads toward gender equity concerning the rights and privileges of young individuals in Nigeria. Utilizing a cross-sectional quantitative approach, data were collected from 605 household heads across six local government areas in Ebonyi State. The findings reveal that 46.32% of male and 62.81% of female heads disagreed with the statement, “a good woman never questions her husband's opinions, even if she is not sure she agrees with them.” Additionally, male heads with the highest wealth index were 10.46 times more likely to have a positive attitude toward the rights and privileges of young girls compared to female heads. The study underscores the necessity of engaging household heads in interventions aimed at transforming beliefs about gender equity to enhance the health and wellbeing of young people.

Ganesan and Gopalakrishnan's study explores the lived experiences of teenage girls and boys who endured sexual abuse during the COVID-19 pandemic in Chennai, India. Utilizing interpretative phenomenological analysis, the researchers conducted interviews to delve into the spatial, corporeal, and temporal aspects of these experiences. The findings highlight the significant role of material and cultural contexts in the occurrence of sexual abuse and identify gaps in existing intervention mechanisms. The study advocates for enhanced prevention strategies that consider the material world and align with Sustainable Development Goals 5 and 16, aiming to promote justice, peaceful societies, and gender equality.

Athanasiades et al. investigate the prevalence and impact of various sexually harassing behaviors among university students, focusing on the influence of gender and psychological resilience. The research involved 2,134 students (70.5% women), both undergraduates (81%) and postgraduates (19%), who completed an online questionnaire assessing experiences of sexual harassment, its consequences, and levels of resilience. Findings indicate that offensive sexual comments, inappropriate remarks about one's body or sex life, and obscene gestures were the most common forms of harassment, predominantly affecting female students. Additionally, women reported more pronounced psycho-emotional and academic consequences as a result of such experiences. The study underscores the need for targeted interventions to address sexual harassment in academic settings, considering the differential impacts based on gender and individual resilience levels.

Aran-Ramspott et al. explore gendered perceptions of social media among Spanish adolescents through 14 focus groups (N = 76). The study identifies distinct gender patterns: young women linked Instagram and TikTok with social pressures on body image and fashion, while young men showed less awareness of these influences. Both genders valued social media for fostering belonging, though women noted self-esteem pressures from idealized images, while men associated it with confidence and enjoyment. Both groups expressed concern about these pressures' impact on women's body image. Findings highlight social media's role in reinforcing self-image norms and the need for edu-communicative initiatives on gender inequalities.

The study by Hurtado-Reina et al. examines gender disparities in academic roles within Spain's Primary Education Degree programs. Using a qualitative-comparative analysis of 794 course syllabi, the study evaluates female representation in course leadership, coordination, and bibliographic resources. Findings reveal a predominantly male-centered bibliographic landscape, despite significant female participation in course direction. The research highlights a clear androcentric bias, where women's academic contributions are undervalued. This work underscores the need for systemic changes to ensure gender balance in educational materials and increased visibility of women's scholarly work, advocating for institutional policies that address gender equity in academia.

Estévez et al. investigate the unique characteristics of gambling disorder in women, as the onset age has recently equalized between genders. Using three discussion groups with 18 women aged 30–68 and an inductive coding process via Atlas.Ti 22.0, the research explores gambling motives, preferences, and related pathologies. Findings reveal that childhood abuse, family influence, and maladaptive emotion management are key factors in gambling initiation. Women often choose less visible games due to social stigma. High comorbidity with disorders like depression, anxiety, and substance use is noted. The study calls for more research to address women-specific factors in gambling disorder.

Qin et al. examine the relationship between years of education and subjective wellbeing among Chinese women, using data from the 2021 China General Social Survey. Employing an ordered Logit model and a coupling coordination model, the study finds that more years of education positively impact subjective wellbeing, with stronger effects in economically developed coastal regions. The research reveals that education enhances wellbeing through improved economic status, health, social and personal cognition, and environmental awareness. This study highlights regional disparities in education's impact, suggesting that policy adaptations are essential to address these differences and optimize women's access to educational benefits.

Chela-Alvarez et al. studied workplace bullying and sexual harassment among hotel housekeepers, using a mixed-methods approach including interviews, focus groups, and a cross-sectional survey (*n* = 1,043). Participants tended to normalize lower-severity harassment, perceiving it as intrinsic to their work environment. Qualitative findings reveal reluctance to label such experiences as harassment, especially in less severe cases. Quantitative results indicate a high prevalence of workplace bullying, significantly associated with increased stress, lower job and salary satisfaction, and diminished social support. These findings underscore the importance of interventions to address the normalization of harassment and highlight vulnerabilities in gendered labor within precarious roles.

Etxebarria-Perez-de-Nanclares et al. examined the unsafe school environment for trans individuals, highlighting issues such as discrimination, bullying, and a lack of teacher training. Using narrative research and interviews, they found that binary societal norms and insufficient gender identity content in education negatively impact trans students' experiences. Early transitions are shown to improve socioemotional wellbeing, but access to information is crucial for this. An important conclusion of the study is that it is the school that must undergo a transition to adequately address the needs of transgender children and youth.

Karamitanha et al. investigated intimate partner violence (IPV) against women in Zanjan city, Iran, revealing that 79.7% of participants experienced at least one form of IPV, with psychological violence being the most prevalent (76.6%) and economic violence the least (12%). The study also identified higher violence rates among women with lower household incomes, younger age, and husbands in self-employment. These findings underscore the need for targeted interventions and improved health policies.

Bae and Jeong conducted an online experiment using Video Interventions for Diversity in STEM (VIDS) to assess the effectiveness of shortened video interventions in reducing gender bias among Korean adults. Participants who watched gender bias videos showed significantly lower bias compared to the control group, with logical thinking enhancing the emotional immersion's effect on bias reduction. The study highlights the potential of brief video-based interventions to foster perspective-taking and decrease gender bias in society.

Xue and Xu investigated gender and geographic diversity within editorial boards of education journals, revealing that while gender diversity is relatively balanced (58.62% female editors-in-chief), geographic disparities are pronounced, with 93.52% of editors from developed countries. No correlation was found between impact factor and female representation. The study emphasizes the need for improved geographic diversity to ensure equitable representation from developing countries.

Wen et al. explored how curriculum experience, extracurricular activities, and faculty support influence the self-perceived employability (SPE) of female STEM students in China. The study, using data from 59,066 students, found that these factors positively impact SPE, though women still report lower SPE than men across all university tiers. The research highlights that university stratification affects female students' experiences and career expectations, with men benefiting more from college activities in most areas except academics.

